# Gadolinium free cardiovascular magnetic resonance with 2-point Cine balanced steady state free precession

**DOI:** 10.1186/s12968-015-0194-1

**Published:** 2015-10-29

**Authors:** Tori A. Stromp, Steve W. Leung, Kristin N. Andres, Linyuan Jing, Brandon K. Fornwalt, Richard J. Charnigo, Vincent L. Sorrell, Moriel H. Vandsburger

**Affiliations:** Department of Physiology, University of Kentucky, 741 South Limestone Street, BBSRB room 355, Lexington, 40536 KY USA; Gill Heart Institute, University of Kentucky Healthcare, Lexington, KY USA; Saha Cardiovascular Research Center, University of Kentucky, 741 South Limestone Street, BBSRB room 355, Lexington, 40536 KY USA; Department of Pediatrics, University of Kentucky Healthcare, Lexington, KY USA; Department of Biomedical Engineering, University of Kentucky, 741 South Limestone Street, BBSRB room 355, Lexington, 40536 KY USA; Departments of Statistics and Biostatistics, University of Kentucky, Lexington, KY USA

**Keywords:** Cardiovascular magnetic resonance, Infarction, Cardiomyopathy, Remodeling, Myocardium

## Abstract

**Background:**

Cardiovascular magnetic resonance (CMR) of ventricular structure and function is widely performed using cine balanced steady state free precession (bSSFP) MRI. The bSSFP signal of myocardium is weighted by magnetization transfer (MT) and T1/T2-relaxation times. In edematous and fibrotic tissues, increased T2 and reduced MT lead to increased signal intensity on images acquired with high excitation flip angles. We hypothesized that acquisition of two differentially MT-weighted bSSFP images (termed 2-point bSSFP) can identify tissue that would enhance with gadolinium similar to standard of care late gadolinium enhancement (LGE).

**Methods:**

Cine bSSFP images (flip angles of 5° and 45°) and native-T1 and T2 maps were acquired in one mid-ventricular slice in 47 patients referred for CMR and 10 healthy controls. Afterwards, LGE images and post-contrast T1 maps were acquired and gadolinium partition coefficient (GPC) was calculated. Maps of ΔS/S_o_ were calculated as (S_45_-S_5_)/S_5_*100 (%), where S_flip_angle_ is the voxel signal intensity.

**Results:**

Twenty three patients demonstrated areas of myocardial hyper-enhancement with LGE. In enhanced regions, ΔS/S_o_, native-T1, T2, and GPC were heightened (*p* < 0.05 vs. non-enhanced tissues). ΔS/S_o_, native-T1, and T2 all demonstrated association with GPC, however the association was strongest for ΔS/S_o_. Bland-Altman analysis revealed a slight bias towards larger volume of enhancement with ΔS/S_o_ compared to LGE, and similar transmurality. Subjective analysis with 2-blinded expert readers revealed agreement between ΔS/S_o_ and LGE of 73.4 %, with false positive detection of 16.7 % and false negative detection of 15.2 %.

**Conclusions:**

Gadolinium free 2-point bSSFP identified tissue that enhances at LGE with strong association to GPC. Our results suggest that with further development, MT-weighted CMR could be used similar to LGE for diagnostic imaging.

**Electronic supplementary material:**

The online version of this article (doi:10.1186/s12968-015-0194-1) contains supplementary material, which is available to authorized users.

## Background

Cardiovascular magnetic resonance (CMR) has become a reference standard modality to image ventricular structure, contractile function, and perfusion [1]. Combined with intravenous administration of gadolinium contrast agents, late gadolinium enhancement (LGE) CMR has become the standard of care to identify myocardial edema, necrosis, and focal fibrosis. The presence of LGE correlates with significantly increased risk of adverse cardiac events and mortality [2]. Recent studies that identify diffuse fibrosis through measurement of gadolinium partition coefficient (GPC) or the extracellular volume fraction (ECV) [3, 4] have similarly demonstrated a strong correlation between diffuse fibrosis and increased mortality [5]. However, residual concerns surrounding gadolinium and nephrogenic systemic fibrosis [6] have spurred the development of gadolinium-free methods to identify diseased myocardium.

Both edematous and fibrotic myocardium are characterized by an increased extracellular volume fraction, which results in lengthened native-T1 and T2-relaxation times compared to healthy myocardium. These changes have been used to identify edema in acute MI [7, 8] and fibrosis in select cardiomyopathies [9–11]. Recent studies using native T1-mapping to identify fibrosis are highly promising [12–14]. However, measured myocardial T1-relaxation times vary between T1-mapping pulse sequences [15] and myocardial regions [16], require special sequence modifications to reduce arrhythmia sensitivity [17], and reconstruction of T1-maps requires motion correction [18] that has limited some prior measurements to the septum [9, 11, 19, 20]. In contrast, cine balanced steady state free precession (bSSFP) is ubiquitously used to image ventricular structure and function. While weighting of the bSSFP signal by a factor of √T2/T1 is established, modulation of the bSSFP signal by magnetization transfer (MT) from extracellular matrix macromolecules has only recently been understood [21, 22]. Specifically, myocardium characterized by increased ECV demonstrates reduced MT compared to healthy myocardium, as demonstrated in a prior study of acute-MI [21]. However, whether MT-weighted CMR can be used to identify tissues that would enhance with gadolinium across a range of cardiomyopathies similar to LGE has not been examined.

We hypothesized that acquisition of bSSFP cine image sets with different MT-weighting (termed 2-point bSSFP) could combine the changes in signal intensity due to both lengthened T1/T2-relaxation and reduced MT to identify tissue that would enhance with gadolinium in close agreement to LGE. We compared tissue characterization with 2-point bSSFP, native-T1 and T2-mapping to LGE in 47 patients referred for CMR at our institution. Our results demonstrate robust agreement between gadolinium free 2-point bSSFP imaging and standard of care LGE, with a strong association between 2-point bSSFP and GPC.

## Methods

### Patient selection, ethics, consent and permissions

Fifty non-consecutive patients referred for clinically indicated CMR with gadolinium contrast were prospectively enrolled, however 3 were excluded due to inability to maintain breath-holds. All patients referred for CMR with gadolinium contrast at our institution over a six month period were approached for study participation, with the forty seven included in the study representing those that consented to participate. Afterwards, ten healthy age-matched controls were recruited but did not receive gadolinium. The research protocol was approved by our institutional review board (IRB 12-0795-F3R) and informed consent was obtained from all subjects for participation and publication of findings. Demographic characteristics are summarized in Table 1. Clinical CMR reports were used to obtain ejection fraction (EF), end-diastolic volume (EDV), and CMR diagnosis.

**Table 1:** Participant characteristics

Variable	Healthy Control (Group I)	CVD without Enhancement (Group II)	CVD with Enhancement (Groups III, IV)	*p*-value
	(*n* = 10)	(*n* = 24)	(*n* = 23)	
Demographics				
Age (yrs)	51.74 ± 4.7	47.7 ± 16.5	51.39 ± 15.4	.406
BMI (kg/m^2^)	23.32 ± 1.5	29.3 ± 6.7	27.4 ± 3.6	.007
Female	4 (50.0)	8 (33.3)	4 (17.4)	.315
White	9 (90.0)	20 (83.3)	18 (78.3)	.815
African American	0	3 (12.5)	3 (13.0)	1.00
Hispanic or Other Race	1 (10.0)	1 (4.2)	2 (8.7)	.051
CMR Indication				
Cardiomyopathy		7 (29.2)	10 (43.5)	.371
Hypertrophic Cardiomyopathy		2 (8.3)	1 (4.3)	1.000
Pericarditis, Myocarditis		2 (8.3)	2 (8.7)	1.000
Sarcoidosis		2 (8.3)	2 (8.7)	1.000
Syncope		4 (16.7)	0	.109
Viability		3 (12.5)	5 (21.1)	.461
Other		4 (16.7)	3 (17.4)	1.000
Diagnosis				
Ischemic Cardiomyopathy		4 (16.7)	10 (43.5)	.060
Non-Ischemic Cardiomyopathy		10 (41.7)	7 (30.4)	.547
Hypertrophic Cardiomyopathy		0	2 (8.7)	.234
No Evidence of Cardiomyopathy		8 (33.3)	0	.416
Other		2 (8.3)	4 (17.4)	.416
Ejection Fraction (%)		50.13 ± 14.4	42.57 ± 14.6	.081
End Diastolic Volume (mL)		190.6 ± 76.0	217.65 ± 81.1	.244

CVD without Enhancement: Patients referred for CMR not demonstrating LGE enhancement in imaged slice

CVD with Enhancement: Patients referred for CMR demonstrating LGE enhancement in imaged slice

*BMI*: Body Mass Index (kg/m^2^)

### Cardiac MRI protocol

CMR was performed on a 1.5 T Siemens MAGNETOM Aera scanner (Siemens Medical Imaging Solutions, Erlanger, Germany) using an 18 channel body coil and 12 channel spine coil. A short-axis stack of bSSFP cine images were obtained with prospective ECG triggering to cover the entire heart (TE: 1.2 ms TR: 3.2 ms, bandwidth: 930Hz, field of view: 260x260mm, slice thickness:8 mm, flip angle: 50°, 256x256matrix, GRAPPA 2), from which one mid-ventricular slice was identified for further imaging. The signal intensity of bSSFP images acquired with high excitation flip angles and short repetition times is heavily weighted by MT, T1 and T2, while identical images acquired with low flip angles reflect proton density weighting with minimal contributions from MT. In the identified slice, pairs of bSSFP cine images were acquired with excitation flip angles of 5° (proton density reference) and 45° (MT,T1,T2-weighted) during end-expiratory breath-holds. Native myocardial T1-relaxation times were assessed using a modified Look-Locker imaging (MOLLI) sequence (5(3)3, TE: 1.1 ms, TR: 2.7 ms, flip angle: 35°, bandwidth: 1085Hz, field of view: 272x 272 mm, slice thickness: 8 mm, 256 matrix with 66 % phase resolution, partial Fourier transform 7/8, GRAPPA 2). T2-relaxation times were assessed using a gradient echo readout (T2 preparations of: 0 ms, 25 ms, 55 ms with 3 heart beat recovery in between, TE:1.1 ms, TR:3.2 ms, bandwidth: 1184Hz, field of view: 272 × 272 mm, slice thickness:  8mm, 192 matrix with 75 % phase resolution, partial Fourier transform 6/8, GRAPPA 2) in the same short axis slice during diastasis. Afterwards, gadolinium (0.2 mmol/kg Gd-DTPA) was administered intravenously as a bolus (rates ranged from 2 ml/s to 5 ml/s) and after 15 min LGE images were obtained using segmented gradient recalled echo inversion recovery (TE: 3.2 ms, TR: 8.3 ms, flip angle: 25°, Bandwidth:140Hz) with inversion time set to optimally null the myocardium. Finally, post-contrast MOLLI (4(1)3(1)2, TE:1.1 ms, TR:2.7 ms, flip angle: 35°, field of view: 272 x 272 mm, GRAPPA 2) images were obtained in the same slice position as pre-contrast images. Normal volunteers only underwent non-contrast portions of the protocol.

### Image analysis

Maps of T1 and T2-relaxation times were automatically reconstructed after motion correction using non-rigid body correction. The reproducibility of breath-hold position and the degree of mis-alignment between 5° and 45° scans was assessed via calculation of the DICE similarity coefficient for both complete images and segmented images in which only the heart was included. 2-point bSSFP data was analyzed by calculating the normalized change in signal between images as (ΔS/S_o_)_i_ = [(S_45_-S_5_)/S_5_]_i_, where S_45_ and S_5_ represent the signal intensity for 45° and 5° excitations respectively for each cardiac phase (*i*). For each patient ΔS/S_o_ maps from three diastolic phases without cardiac motion were averaged together to reduce random noise. Maps of GPC were calculated as GPC = (ΔR_1,myocardium_/ΔR_1,blood_) from reconstructed T1-maps.

Data from patients receiving gadolinium were divided and analyzed in a double-blinded manner. An SCMR level-III reader (SWL) used a custom designed MATLAB script to segment the myocardium and define a non-enhanced region of interest (ROI) in each LGE image. Myocardial voxels with signal intensity greater than 5 standard deviations (SD) above the mean of the defined ROI were classified as enhanced at LGE. Maps defining LGE-enhanced and non-enhanced regions were saved, transmitted to MHV, and used to segment ΔS/S_o_, native-T1, T2, and GPC maps. To avoid partial volume errors and account for minor differences in spatial resolution, endocardial and epicardial borders were slightly adjusted to remove the blood pool and pleural space. Measurements in healthy controls and patients without LGE-enhancement were performed over all voxels in the myocardium.

In data acquired from patients demonstrating enhancement at LGE, the enhanced area was calculated as the percentage of all myocardial voxels classified within the enhanced ROI. To calculate the enhanced area from maps of ΔS/S_o_, a threshold value of 197 % (representing the mean + 3 standard deviations of the mean from the healthy control cohort) was applied and used to calculate the fraction of myocardial voxels above the threshold. Transmurality was calculated as the percentage of enhancement along the radial direction at the center of the area of enhancement for LGE and ΔS/S_o_ maps.

**Fig. 1:**
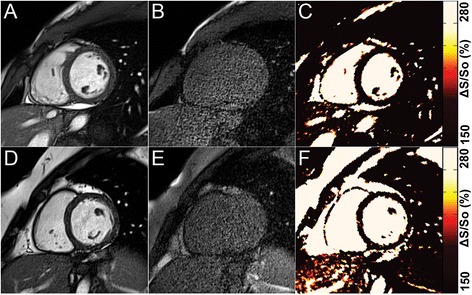
**a-c** Representative data from a healthy control. End-diastolic reference bSSFP images acquired with (**a**) 45° and (**b**) 5° flip angles provide MT-weighted and proton density reference images, respectively. (**c**) Maps of ΔS/S_o_ that are calculated from A and B demonstrate uniform and low values throughout the heart. **d-f** Representative data from a patient without LGE. End-diastolic reference bSSFP images acquired with (**d**) 45° and (**e**) 5° flip angles. This patient demonstrated no myocardial enhancement at LGE. (**f**) Map of ΔS/S_o_ demonstrates uniformly low values similar to the healthy control. For all maps, the color scale was chosen to emulate LGE imaging, with areas of edema/fibrosis demonstrating signal enhancement and areas of healthy tissue appearing dark

**Fig. 2:**
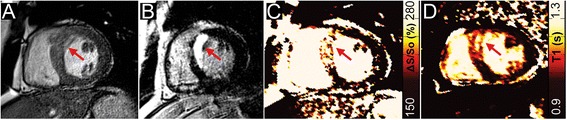
Identification of edema and necrosis in a patient with acute myocardial infarction. (**a**) End-diastolic reference image of a midventricular slice in which 2-point bSSFP, native T1-mapping, and LGE data were acquired. (**b**) LGE imaging reveals an area of hyper-enhancement along the septum indicative of edema and/or necrosis (*red arrow*). The corresponding maps of (**c**) ΔS/S_o_, and (**d**) native-T1 both demonstrate similar spatial patterns of elevated values to LGE (*red arrow*). The corresponding T2-map and windowed bSSFP image can be found in Additional file 1: Figure S2

**Fig. 3:**
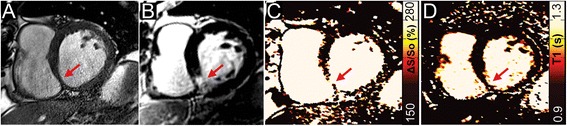
Identification of scar tissue in chronic myocardial infarction. (**a**) Magnitude reconstructed bSSFP image reveals a thinned wall along the inferior right ventricular insertion point (*red arrow*). (**b**) LGE imaging confirms the presence of primarily sub-endocardial scar tissue as an area of signal enhancement (*red arrow*). Mapping of (**c**) ΔS/S_o_ and (**d**) native-T1 both reveal increased values within the scar tissue (*red arrows*), and normal values throughout the remaining myocardium

**Fig. 4:**
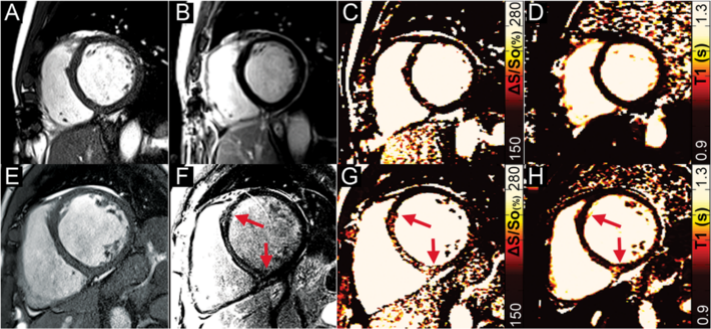
Two patients with non-ischemic dilated cardiomyopathy. (**a, e**) Dilation of the left ventricle is present in both patients on end-diastolic images. In the first patient, (**b**) no LGE-enhancement is present, (**c**) ΔS/S_o_ is normal throughout the heart as is (**d**) native-T1. In the second patient, (**f**) mid-wall septal LGE-enhancement is present (*red arrows*). (**g**) Heightened ΔS/S_o_ is observed in close agreement with the LGE image (*red arrows*), however (**h**) native-T1 values are elevated primarily at the right ventricular insertion-point

Figures were prepared using a median filter with a 3x2 kernel (unfiltered maps can be found in the data Additional file 1: Figure S1). The color scheme for maps of ΔS/S_o_ and native-T1 in Figs. 1, 2, 3, and 4 have been designed to emulate LGE, with non-enhanced tissue appearing dark, enhanced tissue appearing bright, and tissue that would demonstrate diffuse “gray” enhancement appearing red/yellow.

### Subjective assessment by blinded readers

Subjective assessment of 2-point bSSFP in comparison to LGE was performed by two blinded readers with 1 and over 10 years experience. All ΔS/S_o_ maps and LGE images were compiled separately and randomized. The readers were asked to identify the presence, location, and type (focal vs. diffuse) of enhancement, and to delineate the extent of enhancement on each image.

### Statistics

Numeric data are summarized as mean ± SD. For outcome variables we used Version 9.3 of SAS software (SAS Institute, Cary NC) to fit a linear mixed model comparing mean levels across four groups of heart tissue: healthy controls (Group I), patients without LGE-enhancement in the imaged slice (Group II), non-enhanced regions of interest from patients with LGE-enhancement (Group III), and enhanced regions of interest from patients with LGE-enhancement (Group IV). We included random effects for subjects to account for correlations between measurements on non-enhanced and enhanced tissue from the same patient with LGE-enhancement. Linear contrasts were used for pairwise comparisons. Demographic variables were analyzed using SPSS (IBM Corp., 2013). The Shapiro-Wilk method was used to test normality of numeric data. Age, body mass index (BMI), and race were compared across all participants using the Kruskal-Wallis method. Fisher’s exact tests were used to compare gender across all participants and CMR diagnosis between the two patient groups. Differences in EF were compared via Mann–Whitney and EDV was analyzed by student’s *t*-test. Statistical significance in pairwise comparisons was defined by a p-value < 0.05 divided by the number of comparisons to control Type I testing error through Bonferroni adjustment. Otherwise, a p-value < 0.05 defined statistical significance.

## Results

### Demographics and ventricular structure and function

Amongst 23 patients who demonstrated LGE-enhancement in the imaged slice, EDV trended higher and EF trended lower compared to patients who did not demonstrate LGE-enhancement (Table 1). There were no significant differences in age or BMI between patients with and without LGE enhancement. Control participants differed only in BMI compared to patients (*p* < 0.001 for all).

### MR tissue characterization

The DICE similarity coefficient measured across all patients was 0.995 ± 0.004 when comparing entire 5° and 45° images. Comparison of the same images following segmentation of only the heart revealed a DICE similarity coefficient of 0.991 ± 0.015. Representative bSSFP images and maps of ΔS/S_o_ in a healthy control subject and a patient without LGE-enhancement revealed uniformly low ΔS/S_o_ values across both hearts (Fig. 1). In patients with acute (Fig. 2) and chronic MI (Fig. 3), CMR tissue characterization with 2-point bSSFP demonstrated heightened ΔS/S_o_ values in close spatial agreement with LGE-CMR enhancement patterns. Representative images acquired in two patients with non-ischemic dilated cardiomyopathy demonstrate the accurate detection of fibrotic tissue using 2-point bSSFP (Fig. 4). Elevated native T1-relaxation times were also observed in agreement with LGE following MI (Figs. 2 and 3).

**Fig. 5:**
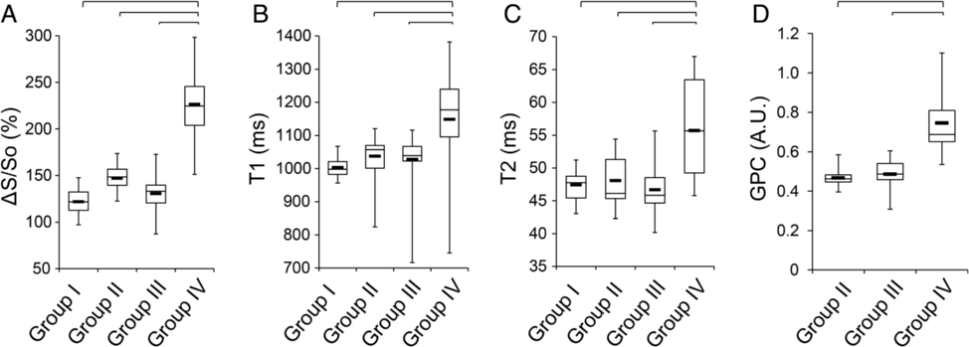
Tissue characterization parameters. (**a**) ΔS/S_o_ was significantly elevated in tissue regions that enhanced on LGE images (Group IV) compared to non-enhanced regions from the same patients (Group III), patients without any LGE (Group II), and healthy controls (Group I). Similarly, (**b**) native-T1 and (**c**) native-T2 were significantly elevated in tissue that enhanced on LGE images compared to all other groups. (**d**) GPC was significantly higher in tissue that enhanced on LGE images compared to non-enhanced tissue regions in patients. (Lines represent *p* < 0.05)

**Fig. 6:**
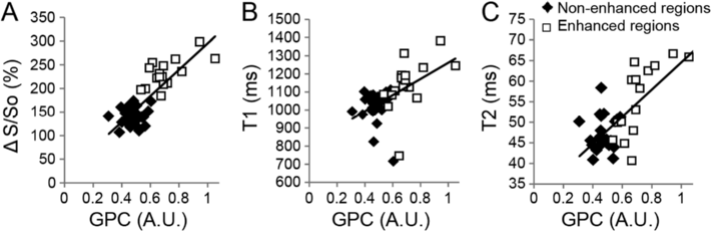
Association of tissue characterization parameters with GPC. (**a**) ΔS/S_o_ (R = 0.82), (**b**) native-T1 (R = 0.55), and (**c**) T2 (R = 0.75) all associated strongly with GPC. Data points are shown for all measurements as either enhanced on LGE images (*white boxes*) or non-enhanced on LGE images (*black diamonds*)

Average myocardial ΔS/S_o_, native-T1 and T2 relaxation-times were significantly higher in LGE-enhanced regions (Group IV) compared to all non-enhanced regions (Groups II and III) and healthy controls (Group I, Fig. 5). The mean of the standard deviation of ΔS/S_o_ values amongst healthy controls was 27.1 ± 8.1 (%) in absolute terms. Segmentation of the heart into twelve equal circumferentially spaced sectors revealed moderately lower average ΔS/S_o_ values (118.8 ± 14.7 (%)) in the anterior-lateral wall compared to the rest of the myocardium. Additionally, GPC was significantly elevated in LGE-enhanced regions (Fig. 5). Native-T1 and T2-relaxation times and ΔS/S_o_ did not differ significantly between non-enhanced myocardium in patients (Groups II and III) and healthy controls (Group I, Fig. 5). Native-T1, T2 and ΔS/S_o_ all demonstrated strong association with GPC (Fig. 6).

**Fig. 7:**
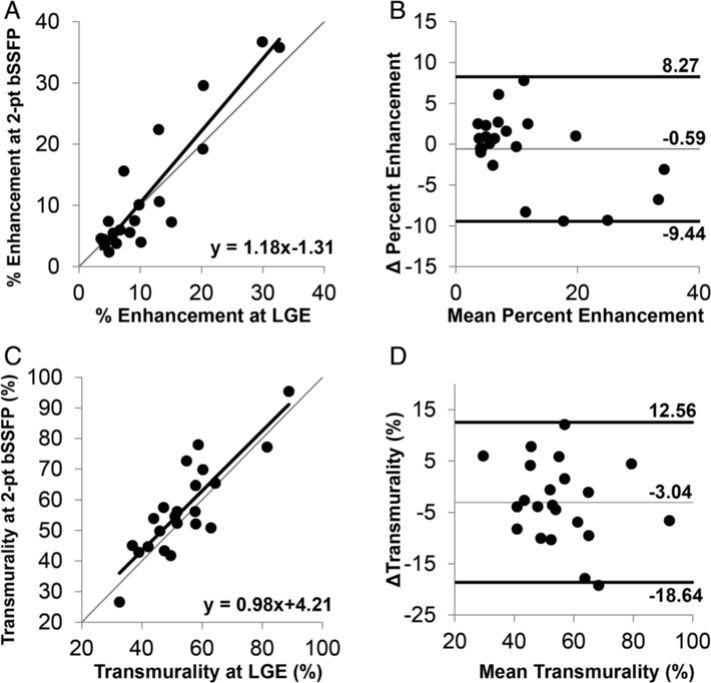
Association of enhanced area and transmurality between 2-point bSSFP and LGE. (**a**) Comparison of the enhanced myocardial area (represented as percent of total myocardial area) using 2-pt bSSFP and LGE revealed a strong association between the two methods (R^2^ = 0.84) with a slight bias towards larger areas of enhancement with 2-point bSSFP. (**b**) Bland-Altman plot comparing the difference between enhanced areas by both methods to the mean between both methods revealed a coefficient of variation of 0.204. (**c**) Similarly, the comparison of the transmurality of enhancement by each method revealed a strong association between 2-point bSSFP and LGE (R^2^ = 0.73) with (**d**) Bland-Altman analysis demonstrating a coefficient of variation of 0.0875

Quantification of the percent of myocardium classified as enhanced at 2-point bSSFP demonstrated a strong association (R^2^ = 0.84) with the percent of myocardium classified as enhanced at LGE (Fig. 7), however a slight bias towards over-estimation of the enhanced area in patients with a higher percentage of enhancement was observed. Bland-Altman analysis (Fig. 7) revealed a coefficient of variation of 0.204. Measurement of the transmurality of enhancement was similar between 2-point bSSFP and LGE (R^2^ = 0.73), and Bland-Altman analysis revealed a coefficient of variation of 0.0875 (Fig. 7).

### Subjective assessment

**Fig. 8:**
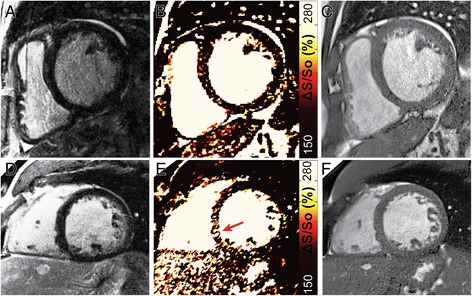
False positive identification of enhancement at ΔS/S_o_. (**a-c**) Scattered noise on ΔS/S_o_ maps led to the false identification of diffuse enhancement in 3 of the 4 false positive cases. A representative example of a patient without enhancement at LGE (**a**) that was classified by blinded readers as demonstrating diffuse enhancement at ΔS/S_o_ (**b**) in the septum with corresponding anatomical reference image (**c**). **d-f** In one patient without enhancement at LGE (**d**), focal enhancement (*arrow*) was identified on the corresponding map of ΔS/S_o_, with corresponding anatomical image shown in (**f**)

**Fig. 9:**
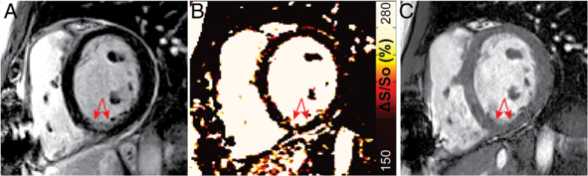
False negative identification at ΔS/S_o_. All cases in which blinded readers identified individuals with enhancement at LGE as normal at ΔS/S_o_ occurred in cases of sub-endocardial enhancement. (**a**) Representative LGE image from a patient with 50-75 % sub-endocardial intermediate signal enhancement (*red arrows*) in the inferior wall. (**b**) The corresponding map of ΔS/S_o_ demonstrates elevated values in the same region (*red arrows*). (**c**) The corresponding anatomical reference image confirms that the elevated ΔS/S_o_ values in (**b**) occur in myocardial tissue

Analysis of ΔS/S_o_ maps and LGE images by 2 blinded readers revealed an average agreement of 73.4 % between methods. Among the patients demonstrating enhancement at LGE, the extent of enhancement on ΔS/S_o_ maps was identified as the same in an average of 67.2 % of individuals. The extent of enhancement was identified as greater in ΔS/S_o_ maps in 20.8 % of individuals, and smaller in ΔS/S_o_ maps in 12.0 % of individuals. An average of 4 out of 24 patients in which enhancement was not identified in LGE images were classified as demonstrating enhancement on ΔS/S_o_ maps (Fig. 8). Among the 23 patients demonstrating enhanced tissue at LGE, an average of 3.5 were classified as normal by readers interpreting ΔS/S_o_ maps (Fig. 9). In all such cases, enhancement patterns were consistent with small sub-endocardial enhancement at LGE.

## Discussion

In this study we present a new 2-point bSSFP method for gadolinium-free CMR. In 47 patients undergoing clinical LGE examination, 2-point bSSFP demonstrated a strong association between elevated ΔS/S_o_ and enhanced regions in LGE across a range of cardiomyopathies. Further, 2-point bSSFP demonstrated similar results to mapping of native-T1 relaxation times. Importantly, in this study we used a relatively simple method to generate MT contrast in bSSFP images. However, MT contrast can be further enhanced through the use of additional MT-preparation schemes, potentially increasing the sensitivity of CMR tissue characterization with MT contrast.

Heightened steady state signal in edematous cardiac tissue occurs in bSSFP images acquired with a short repetition time and high flip angle. In a study by Zhou et al. [22], edematous myocardium was visualized as hyper-intense on bSSFP images following ischemia-reperfusion injury in dogs. By comparison to T2-prepared SSFP images, the authors concluded that edema contrast in bSSFP was dominated by changes in MT and proton density (65 %), with altered relaxation times having a more modest effect (35 %). Similarly, Kumar et al. [23] observed a 50 % increase in bSSFP signal in infarcted tissue in dogs and patients with acute MI. While we observed increased signal intensity in edematous areas on bSSFP images, we found that visualization required significant contrast adjustments and resulted in noisy images (Additional file 1: Figure S2). In 2-point bSSFP, changes in signal intensity on standard bSSFP cine images caused by increased T2 and reduced MT in tissue that enhances at LGE were extracted by normalization to images acquired with a 5° flip angle (proton density weighted). Measurement of ΔS/S_o_, which was elevated in enhanced tissue in patients with acute MI, was consistent with signal intensity changes seen by Kumar et al. [23] and Zhou et al. [22] and demonstrated strong spatial agreement with LGE (Fig. 2). In addition, patterns of heightened ΔS/S_o_ in patients with acute-MI mirrored T2-mapping (Additional file 1: Figure S2), which is widely used to identify edema [7, 8]. Our results further agreed with Weber et al. [21] who demonstrated altered MT-ratio in patients with sub-acute MI by acquiring pairs of bSSFP images with different MT-weighting. In the study by Weber et al. MT-contrast was generated by altering the duration of the RF excitation pulse and the repetition time between cardiac phases causing reduced MT-ratio in edematous tissue in comparison to healthy tissue. However, elongation of the excitation pulse meant that differences in cardiac phase were present in images used to calculate the MT ratio. We chose to change the excitation flip angle, and not duration, in order to have a consistent cardiac phase between MT-weighted and proton density weighted images. Subsequently, our measure of ΔS/S_o_ is heightened in tissues that would enhance with LGE.

Identification of focal fibrosis with LGE is the established clinical standard and in our study heightened ΔS/S_o_ occurred in tissues identified by LGE as replacement (Fig. 3) and reactive fibrosis (Fig. 4). Emerging techniques to image diffuse fibrosis including mapping of post-contrast T1-relaxation times and measurement of GPC or ECV [3, 4] have been correlated to collagen volume fraction at biopsy [24] and demonstrated predictive value for clinically relevant outcomes [5, 25, 26]. In our study we did not have access to hematocrit, however GPC values measured in non-enhanced myocardium agreed with prior studies of healthy tissue [16, 27, 28] and were significantly elevated in regions of interest identified by LGE (Fig. 5). Comparing ΔS/S_o_ to GPC revealed a strong and promising association (Fig. 6). However, detection of diffuse fibrosis with 2-point bSSFP requires further study with a larger sample and a consistent phenotype such as hypertrophic cardiomyopathy. In addition, since bSSFP images are weighted by √T2/T1, increased T1 relaxation times in fibrotic scar tissue will have the opposite effect of decreased MT on the steady state signal in the high flip angle acquisition (Additional file 1: Figure S3). While this may be partially mitigated by concomitant increases in T2 relaxation times (Additional file 1: Figure S3), the balance between increased T1 and reduced MT, and the potential limits this imposes upon detection via measurement of ΔS/S_o_, requires additional examination in a large cohort of patients with chronic MI. In addition, given the contributions of MT, T1, and T2 to ΔS/S_o_, it is unclear whether measurement of specific ΔS/S_o_ values can be used to differentiate edema from fibrosis. Additional studies with larger cohorts of acute and chronic MI patients are necessary to examine this possibility.

Mapping of native myocardial T1-relaxation times is emerging as a highly promising method for gadolinium-free imaging of fibrosis [12, 14, 16]. Recently, several studies demonstrated increased T1-relaxation times in patients with edema [29], aortic stenosis [9], myocarditis [10], and hypertrophic and non-ischemic dilated cardiomyopathies [11]. Native T1-relaxation times measured in our study using a MOLLI acquisition scheme at 1.5 T were comparable to those measured under similar settings [15, 30] and were significantly elevated in enhanced myocardium (Fig. 5). While we observed a strong association between heightened native-T1and GPC, our association was weaker than observed in prior studies [10, 11]. One likely factor contributing to this difference is that unlike most prior studies that focused on patient cohorts with a specific and profound phenotype, we sampled patients with a range of cardiomyopathies and varying degrees of edema or fibrosis. Also, artifacts introduced by motion correction [18] have led many prior studies to restrict data analysis to the interventricular septum [9, 11, 20]. We analyzed myocardium across an entire short-axis slice, defining regions of interest based on LGE patterns. Results from a recent multi-center T1 mapping study demonstrated considerable regional variability in segmental native-T1 values at 1.5 T [16]. Thus, our results likely reflect the influence of both motion correction artifacts on T1-estimation and regional T1 heterogeneity of healthy tissue that were not included in prior studies. Additionally, our scanner was equipped only with a MOLLI acquisition scheme that has demonstrated sensitivity to MT-effects [13], and thus the sensitivity of native T1-mapping may have improved with other mapping methods now available [15], including recently developed arrhythmia insensitive T1 mapping protocols [17].

Images acquired with an excitation flip angle of 5° demonstrate low signal to noise, potentially leading to artificially elevated measurement of ΔS/S_o_. We sought to limit the effect of random noise by averaging over three identical end diastolic phases and applying a median filtering algorithm to reconstructed maps. However, subjective assessment of ΔS/S_o_ maps by two blinded expert readers resulted in the incorrect interpretation of diffuse enhancement in ΔS/S_o_ maps in all but one of the false positive cases (Fig. 8). We chose to use a 5° excitation flip angle in order to maximize the potential difference in MT-weighting between images, however, the acquisition of such images with slightly higher flip angles may present a more promising route to maintaining MT-contrast between image pairs while reducing the presence of voxels with spuriously high ΔS/S_o_ values. Alternatively, future studies could examine MT-weighting without the use of low flip angle acquisitions via various magnetization preparation schemes that encode greater MT-weighting directly into the steady state magnetization. In addition, subjective analysis of ΔS/S_o_ maps by expert readers revealed a propensity to misidentify small sub-endocardial enhancement patterns as blood instead of enhanced tissue (Fig. 9). In future studies, the use of blood signal suppression should be investigated as a mechanism to mitigate false negative interpretation of ΔS/S_o_ maps.

A limitation to our study was that due to time constraints we acquired data in only one slice per patient without prior knowledge of disease status. In several patients, the slice chosen for our study did not demonstrate LGE-enhancement (Group II), however LGE-enhancement was present in other slices. Additionally, limitations on T1 and T2-mapping protocols on our scanner resulted in acquisition of bSSFP images at slightly higher spatial resolution. Consequently, partial volume error is more likely to influence T2 maps, and to a lesser extent T1 maps, than 2-point bSSFP results. Care was taken to adjust boundaries to exclude border pixels affected by partial volume artifacts, however registration of pre and post gadolinium maps was not performed. The sensitivity to B1 inhomogeneity remains a significant concern in cine bSSFP, particularly at higher flip angles. We simulated the bSSFP signal using a range of myocardial relaxation times and excitation flip angles. Based on the results of our simulation, and prior evidence that MT is maximal and constant above excitation flip angles of 30° [21], we chose to use a 45° flip angle in order to minimize the potential effects of B1-inhomogeneity. Also, changes in through-plane motion can modulate steady state behavior in the myocardium. For this reason we chose to focus our analysis on end-diastolic cardiac phases. In addition, the acquisition of two separate end expiratory breath-held scans increases the potential for misalignment between scans. Measurement of the DICE similarity coefficient between image pairs in our study was high, however we benefited from placement of our scans at the end of the non-contrast CMR workup, thus reducing potential misalignment that could occur if such scans were performed at the initiation of the CMR examination. Importantly, while registration algorithms can be used to compensate as they are in T1 mapping protocols, simple image intensity based algorithms would not be effective for registration of images acquired with a 5° excitation flip angle.

## Conclusions

2-point bSSFP utilizes endogenous contrast mechanisms for gadolinium-free CMR imaging. In this study, we demonstrated across a range of patients strong association between 2-point bSSFP and standard of care LGE-CMR. Importantly, since MT-contrast is an endogenous mechanism, the sensitivity to changes in MT-weighting increases with spatial resolution. In addition, MT-contrast can be further increased with MT-preparation schemes not used in this initial study. In contrast, differences in native-T1 between healthy and diseased tissue can not be further increased without increasing the magnetic field strength. With further development, MT-weighted CMR could potentially enable diagnostic imaging similar to LGE CMR without the use of gadolinium.

## Additional file

Additional file 1:
**Supplementary figures.**

## Abbreviations

CMR: Cardiovascular magnetic resonance
LGE: Late gadolinium enhancement
GPC: Gadolinium partition coefficient
ECV: Extracellular volume fraction
MT: Magnetization transfer
bSSFP: balanced steady state free precession
EF: Ejection fraction
EDV: End diastolic volume
TE: Echo time
TR: Repetition time
MOLLI: Modified Look Locker imaging
ROI: Region of interest
SD: Standard deviation
BMI: Body mass index
RF: Radio frequency
