# Supplementary material for: Gadolinium free cardiovascular magnetic resonance with 2-point Cine balanced steady state free precession
Source: J Cardiovasc Magn Reson. 2015 Oct 29;17:90. doi: 10.1186/s12968-015-0194-1 (PMC4628395; doi:10.1186/s12968-015-0194-1)

**Figure S1.** Unfiltered maps of ΔS/S_o_ corresponding to maps shown in figures 1-4.

**
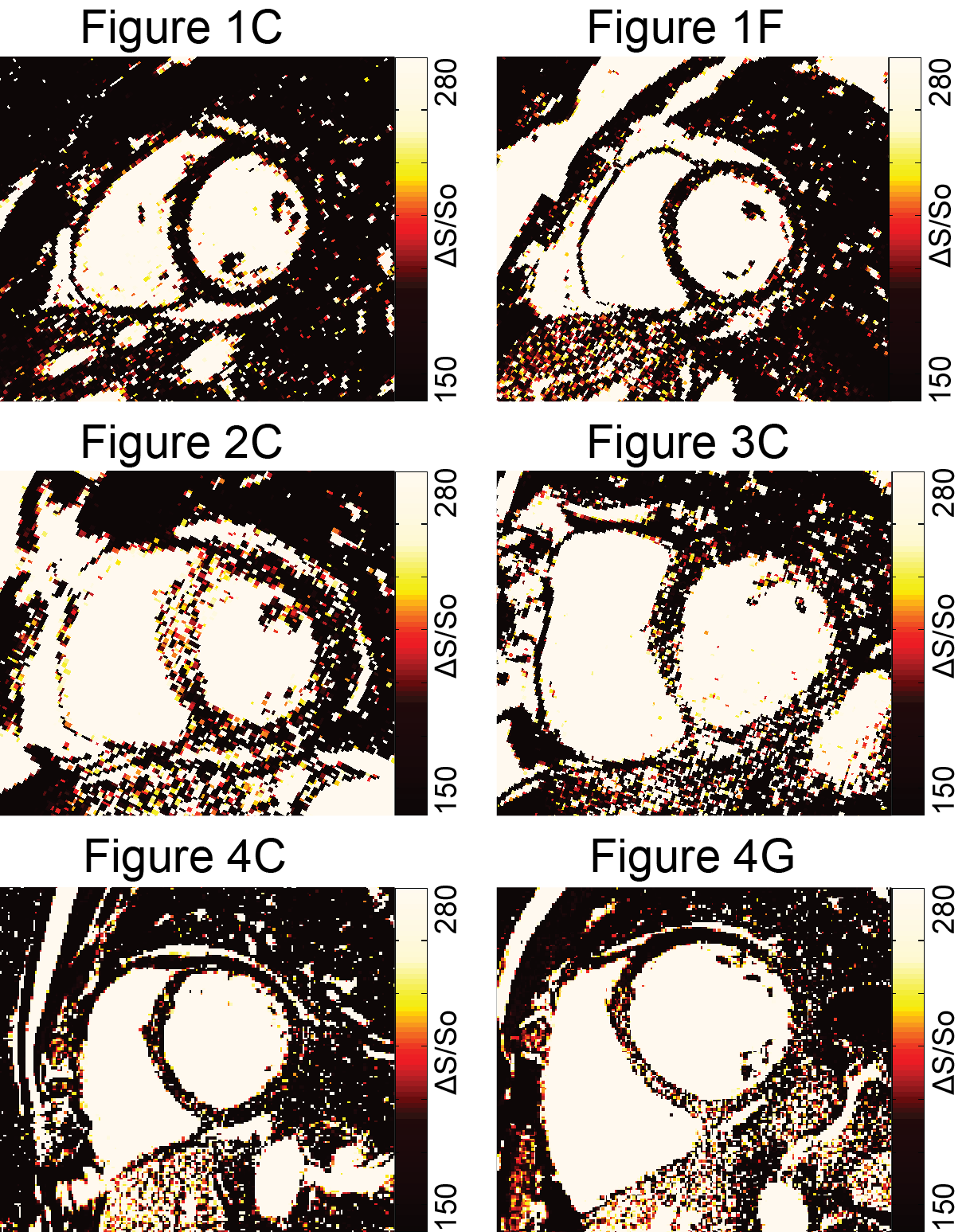
**

**Figure S2. T2-Mapping and Weighting in Acute MI. (A)** Anatomical reference image from same patient as shown in Figure 2, with **(B)** corresponding LGE image demonstrating area of edema following acute MI (red arrow). **(C)** T2-mapping revealed focally and significantly elevated T2 in the area of acute MI, corresponding to edema. **(D)**. Significant contrast manipulation through window and leveling of (A) reveals similar enhancement in the area of edema (red arrow), however scattered noise is seen in areas outside of the enhanced area.


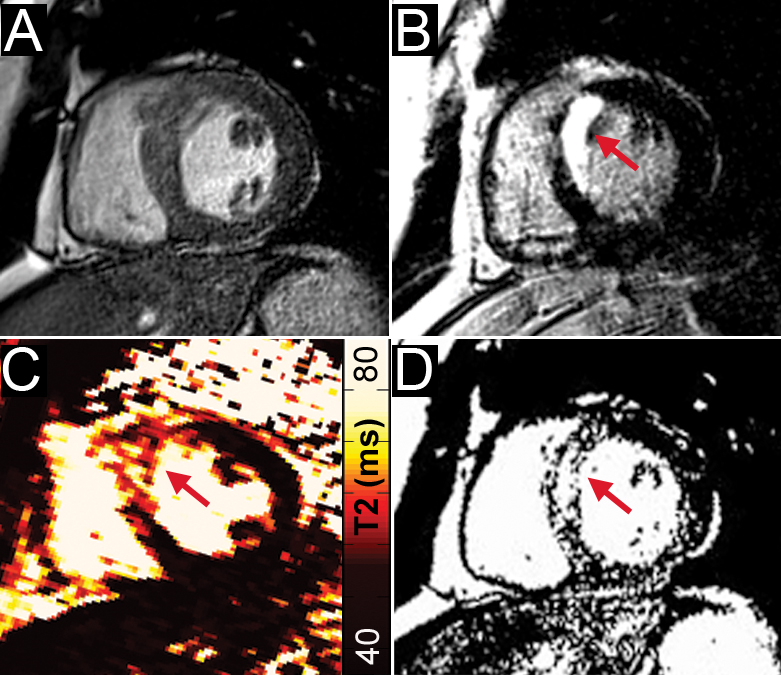


**Figure S3. Simulations of changes in steady state signal intensity as a function of T1, T2, and flip angle.** In order to further examine the impact of changes in relaxation times on the steady state signal intensity, a series of simulations of the steady state magnetization using the closed form equation for cine bSSFP were performed in Matlab. For all simulations we assumed that proton density is 1. **(A)** Using a T2 relaxation time of 48ms (the mean amongst our healthy control cohort), and the TR and TE values from our scanning protocol, simulations confirm that lengthening of T1 relaxation times leads to a reduction in the steady state signal over a physiology relevant range of T1 relaxation times. **(B)** However, changes in T1 relaxation times in fibrotic tissue are often accompanied by changes in T2 relaxation times that can have the opposite effect on the steady state signal. For further simulation of the steady state signal across a range of flip angles we examined healthy values (blue) using the mean T1 (1022.2 ± 48.2 (ms)) and T2 (48.2 ± 2.3 (ms)) relaxation times from our healthy cohort and previously mentioned TR/TE and proton density values. Separately, we simulated fibrotic tissue (red) using the mean T1 and T2 relaxation times from tissues that enhanced at LGE in patients that did not have acute-MI. This population included those with chronic MI and patients with enhancement determined by our cardiologist to be non-ischemic in origin. In this subset of patients (n = 12), the mean native-T1 relaxation time was 1125.6 ± 136.5 (ms), and the mean T2-relaxation time was 52.2 ± 7.7 (ms). Examination of panel B demonstrates that decreases in steady state magnetization due to increased T1 are partially mitigated by increased T2 in fibrotic tissues. **(C)** The difference in steady state signal between the two populations suggests that the contrast observed in maps of ΔS/S_o_ is likely heavily influenced by changes in MT. However, a quantitative understanding of the specific balance between MT and T1 in fibrotic tissue requires further examination in a larger cohort with selection specifically for chronic MI.


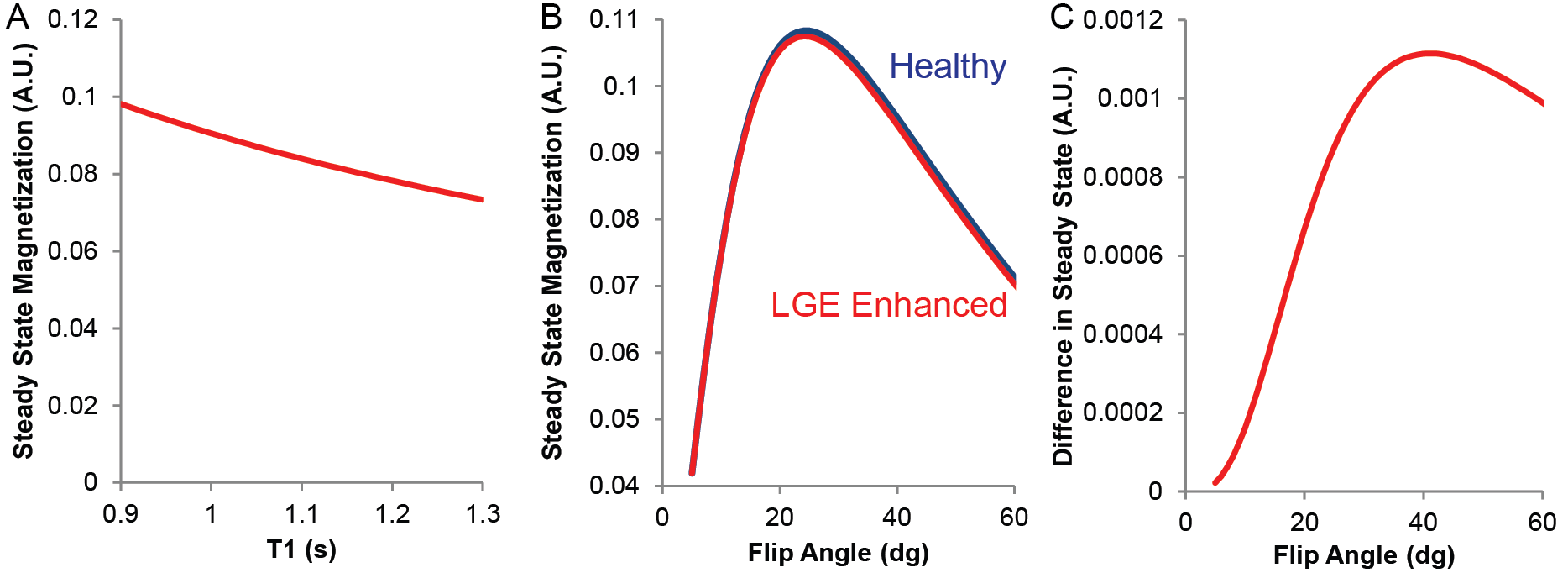

Supplement: Additional file 1: — Supplementary figures. [file 12968_2015_194_MOESM1_ESM.docx]
